# Inhibiting NHEJ: In-Silico Approach to Stratifying DNA-PK Inhibitors, Predicting Radio-Halogenation Potential of DNA-PK Inhibitors, and Assessing AlphaFold2 Prediction Accuracy

**DOI:** 10.3390/bioengineering13050571

**Published:** 2026-05-18

**Authors:** Dragoș Andrei Niculae, Florin-Vlad-Gabriel Crișu, Sabin Stoian, George Nicolae Daniel Ion, Doina Drăgănescu

**Affiliations:** 1 Faculty of Pharmacy, “Carol Davila” University of Medicine and Pharmacy, 37 Dionisie Lupu Street, 020021 Bucharest, Romania; florin-vlad-gabriel.crisu2023@stud.umfcd.ro (F.-V.-G.C.); sabin.stoian2024@stud.umfcd.ro (S.S.); doina.draganescu@umfcd.ro (D.D.); 2 Horia Hulubei National Institute for Physics and Nuclear Engineering, 30 Reactorului Street, 077125 Magurele, Romania

**Keywords:** DNA protein kinase, molecular docking, molecular dynamics, auger electrons, alpha-therapy, Astatine-211, Iodine-123, AlphaFold2, virtual-screening, radiolabeling

## Abstract

Background: The current study integrates AlphaFold2-based structural modeling of DNA-PKcs with an experimentally resolved DNA-PKcs structure, large-scale molecular docking of over 1500 known DNA-PK inhibitors, aiming to identify clinically relevant candidates for next-generation pharmacological delivery methods, evaluate receptor model suitability, and predict how potential radio-halogenation may influence binding behavior and translational radiotherapeutic potential of current clinically relevant candidates. AlphaFold2-based prediction of the DNA-PKcs structure was the starting point, followed by comparison of the AI-derived model with an experimentally resolved structure to evaluate the model’s accuracy and docking suitability. A large database of 1369 known DNA-PK inhibitors was then screened by molecular docking, after which six clinically relevant compounds were analyzed for molecular dynamics. Finally, a focused docking study was conducted on 67 possible radio-halogenated derivatives derived from these clinically relevant scaffolds to investigate the effect of potential iodination, bromination, or astatination on binding behavior. These findings indicate that large-scale docking can be used to comparatively rank DNA-PK-targeting compounds and explore the impact of theoretical radiohalogenation on predicted binding behavior. However, the results also underline the limitations of current receptor models and molecular simulation workflows, supporting the need for experimental validation before translational conclusions are drawn.

## 1. Introduction

Molecular docking is a standard component of structure-based drug discovery, being widely used to prioritize ligands according to their predicted binding mode and estimated interaction strength within the protein binding site [1]. In practice, docking is most useful as a tool for screening and generating hypotheses regarding interaction with the targeted protein, allowing for rapid triage of large compound sets before experimental validation. Its main applications remain pose prediction, virtual screening, and comparative ranking of screened ligands, while its limitations in accurately reproducing full binding thermodynamics require further structural validation [2,3,4,5].

Recent advances in AI-based protein structure prediction have expanded the range of targets accessible to structure-based workflows. Deep learning tools like AlphaFold2 can generate highly accurate structural models directly from amino acid sequence, becoming an important source for structural hypotheses, particularly when experimental coordinates are unavailable, incomplete, or difficult to use directly [4]. At the same time, AI predictions must still be interpreted in context, as local inaccuracies, uncertain loop conformations, and limited representation of ligand-induced or state-dependent structural changes may influence docking performance [6]. When both predicted and experimentally determined structures are available, comparing their behavior within the same docking workflow may provide useful information regarding receptor-model suitability.

In the present study, we focused on DNA-dependent protein kinase (DNA-PK), more specifically, its catalytic subunit (DNA-PKcs), as a target for structure-based design. DNA-PK is the central kinase of the non-homologous end joining (NHEJ) pathway, responsible for repairing radiation-induced DNA double-strand breaks (DSBs) [7,8]. As NHEJ is one of the fastest DSB repair routes employed after ionizing radiation damage [9], tumors that heavily rely on it can attain resistance to radiotherapy [8,10]. As such, inhibition of DNA-PK can impair this early repair response, increase persistence of unrepaired DNA damage, and enhance radiation sensitivity, making DNA-PK inhibitors of particular interest as radiosensitizing agents [11].

It was clinically shown that raised Ku70/DNA-PKcs expression associates with poor tumor control post-radiotherapy [8,12]. Inhibiting DNA-PK inactivates this primary fast-acting repair mechanism, forcing cells to rely on slower or ill-functioning mechanisms [13], homologous recombination, and ultimately overloading its capacity, causing the accumulation of unresolved DSBs and mitotic catastrophe [14,15]. As it was shown that either XRCC4 loss or DNA-PK inhibition via wortmannin stimulates RAD51 formation around DSBs, blocking the DNA-PK should make the cell rely on the much slower homologous recombination (HR) pathway to process DNA damage [16]. With this strategy in mind, potent small-molecule DNA-PK inhibitors have lately been developed and are actively being tested as radiosensitizers in cancer therapy [8,17,18,19]. Detailed mechanisms of NHEJ and the rationale of using DNA-PK inhibitors as part of a multi-approach strategy for cancer radiotherapy are explored in more detail in our published review [8].

Apart from cancer treatment, NHEJ is also a critical component in resolving bacterial and viral infections [20,21]. In infections, DNA-PK has two main roles: sensing the pathogen DNA in order to trigger innate immunity and repairing pathogen-induced DSBs. DNA-PKcs has the ability to bind cytosolic viral DNA, thus activating IRF3/TBK1 and NF-κB pathways to induce type I IFNs and inflammatory cytokines. In contrast, bacteria, such as *H. pylori*, can induce DSBs via a type IV secretion system and host nucleases, and it was shown that these bacterial-induced DSBs are then repaired by the NHEJ machinery via an upregulation of NF-κB target genes [22]. DNA-PK senses the microbial DNA and amplifies cytokine signaling, while NHEJ repairs infection-driven breaks via Ku/DNA-PKcs/XRCC4/LigIV complexes and could introduce mutations and drive oncogenesis.

Several DNA-PK inhibitors have already emerged as representative compounds in the cancer treatment field, with different degrees of selectivity, clinical maturity, and translational relevance. Table 1 lists six of the most representative DNA-PK inhibitors, notable examples including NU7441 (KU-57788), an imidopiperidine that inhibits DNA-PK, and peposertib (M3814), which targets the DNA-PK ATP-binding site with high affinity. Other next-generation agents, such as CC-115, which has a dual inhibition profile, both on DNA-PK and the mTOR pathways, and the selective DNA-PK inhibitor AZD7648, also exhibit sub-100 nM affinity. In preclinical models, these inhibitors potently sensitize tumor cells to ionizing radiation-induced damage, being considered among the most promising radiosensitizing strategies for precision radiotherapy.

In order to understand, predict, and stratify the binding behavior of DNA-PKcs inhibitors, we performed a virtual screening using local molecular docking on a panel of 1369 DNA-PK compounds with known in vitro activity. Docking simulations were carried out using the YASARA Structure software, which integrates AutoDock Vina with explicit-solvent molecular dynamics (MD) refinement for binding positions in order to properly model flexible protein–ligand interactions [23]. Considering the actual technical capabilities, MD analysis was performed only on the already mentioned six clinically relevant DNA-PK inhibitors, with the plan to analyze the rest of them in the future.

Critically, in silico modeling studies can predict how small modifications in a molecule’s structure may impact its behavior, such as affinity to the target, logD, etc. The potential of these DNA-PK inhibitors as future target vectors for pinpoint accumulation in close proximity to the DNA, in the context of targeted radiotherapy, is undeniable. Additionally, a theoretical synergistic effect of inducing DSBs while concurrently inhibiting the repair capacity needs to be assessed in the future. Thus, predicting which compounds future research should focus on can save time, funds, and accelerate the potential clinical translation.

In parallel, we utilized an AI-based protein-modeling program (AlphaFold2) to generate the DNA-PKcs structure based on its amino acid (AA) sequence. We also want to assess the accuracy of AlphaFold2’s prediction, both in terms of modeled protein structure as well as its functionality, represented by docking and MD analysis results [6]. We aim to accurately predict both binding affinity and kinetics for the studied DNA-PK inhibitors by using both advanced docking and in silico MD simulation tools, as well as compare the AI-generated protein with the already experimentally defined one, to assess the program’s reliability for structural modeling.

After completing an initial virtual screening campaign of more than known DNA-PK-targeting ligands, we deliberately narrowed down the following molecular dynamics phase to a clinically anchored, literature-relevant inhibitor panel. Specifically, we selected the six DNA-PK inhibitors (Table 1) highlighted as the relevant selection of DNA-PK inhibitors for clinical precision-radiotherapy approaches, further discussed in our review [8], NU7441, M3814, CC-115, AZD7648, VX-984, and PIK-75 HCl, which also report their core target profiles and representative IC_50_ values. These compounds show highly selective DNA-PKcs ATP-pocket inhibition (M3814, AZD7648, VX-984) as well as multiple-target activity alongside DNA-PK: CC-115 (DNA-PK/mTOR) and PIK-75 (PI3K p110α/DNA-PK). This diversity enables MD refinement on a small yet varied set that remains directly relevant to translational radiosensitization.

From this six-compound panel, we moved forward with in silico analysis of the impact a potential radio-halogenation will have on our compounds, observing how the binding dynamics change after adding one Br, I, or At atom in plausible positions. Docking the halogenated analogs of these three molecules is therefore a rational, hypothesis-driven way to anticipate how these modifications, at chemically viable ring positions, could alter binding geometry and predicted affinity even before planning the synthesis and radiolabeling procedures.

As for the isotope selection, we focus on relevant emission types for a close proximity to DNA accumulation and potential for direct labeling techniques. We looked at direct C-Br, C-I, or C-At introduction strategies that are compatible with small-molecule scaffolds and can be emulated by docking methods. Radioiodination of aromatic small molecules is commonly achieved via electrophilic routes; a widely used practical strategy is iododestannylation—replacement of an aryl-SnR_3_ precursor by radioiodine, in the presence of an oxidant and radioiodide ([^x^I]NaI) [24]—or copper-mediated iododeboronation of a boronic pinacol ester precursor [25]. The main drawback of radioiododestannylation is the precursor toxicity and the formation of additional by-products [26].

Radiobromination of organic scaffolds has also historically been performed through electrophilic substitution using activated radiobrominating species, a strategy most suitable for electron-rich aromatic systems. Although effective, this approach is often limited by substrate scope and reduced compatibility with structurally complex or electron-deficient drug-like molecules. Alternative precursor-based strategies, particularly halodestannylation of organotin intermediates, have therefore been developed to improve regioselectivity and radiochemical efficiency, at the expense of organotin precursor handling and purification complexity. More recently, copper-mediated radiobromination of aryl boronic acids and boronic pinacol esters has emerged as a particularly attractive method, enabling the incorporation of cyclotron-produced bromine radionuclides such as ^77^Br into pharmaceutically relevant (hetero)aryl systems under relatively mild conditions. These newer deboro-bromination strategies considerably broaden the synthetic accessibility of radiobrominated small molecules and are therefore especially relevant for structure-guided development of targeted radiopharmaceuticals.

For Astatine-211, modern radiolabeling chemistry is explicitly described as being translated from radioiodine methods. The field has been dominated by electrophilic astatodemetallation of aryl organometallic precursors, especially trialkylstannyl derivatives, a strategy largely adapted from classical radioiodination chemistry. This approach consists of electrophilic ^211^At species being generated, reacting with aryl stannanes to generate astatinated arenes under relatively mild conditions. This method has long been considered the reference route for preparing ^211^At-labeled small molecules and prosthetic groups. Despite its synthetic utility, astatodestannylation presents several drawbacks, including the need for toxic Sn precursors, purification burdens, and occasional in vivo instability of the C–At bond, depending on the aromatic framework and substitution pattern. Electrophilic astatodemetallation uses trialkylaryltin precursors and oxidants such as chloramine-T or Iodogen^®^ to generate reactive electrophilic halogen species [27]. Recently, alternative electrophilic astatination routes have been developed to address these limitations, including copper-mediated astatination of aryl boronic esters, which enables rapid room-temperature labeling of aryl and heteroaryl substrates without the need for organotin chemistry and trimethylgermyl-based precursors, which have also shown efficient electrophilic ^211^At incorporation across both electron-rich and electron-poor arenes [28,29].

In parallel with precursor-based approaches, the broader ^211^At radiochemistry field is starting to increasingly emphasize the importance of label stability and substrate-specific optimization, since astatine does not always behave as just a heavier iodine analog. Contemporary reviews therefore describe an expanding toolbox that includes both electrophilic and nucleophilic astatination strategies, as well as the development of tailored prosthetic groups and structurally stabilizing motifs intended to improve the in vivo robustness of the astatine label. This growing diversity in labeling strategies is particularly relevant for small-molecule radiopharmaceutical development, where late-stage labeling, preservation of target affinity, and resistance to deactivation are all essential design criteria [30].

Among radionuclides viable to direct incorporation via covalent routes into small-molecule scaffolds, radiohalogens remain the most practical option. In addition to ^125^I, both ^123^I and ^77^Br are relevant Auger electron emitters, with ^77^Br representing a particularly attractive alternative due to its cyclotron production route, higher reactivity with substrates, and its potential for a complete theranostic approach in combination with ^76^Br as an imaging agent, while ^77^Br even has an imageable gamma-line of 238.98 keV (23.1%) [31,32].

The isotope choice then follows radiobiological reasons. Auger emitters are most lethal when the decay occurs as close as possible to critical cellular components, such as the DNA, mitochondria, or cellular membrane, especially if the isotope can be incorporated into DNA [33]. This highlights the theoretical potential of using DNA-PK inhibitors as carriers for Auger/Conversion electron-emitting isotopes, considering that the molecular modifications are small and their impact on selectivity and affinity to their target is kept minimal. Thus, the nuclidic toolbox gets quite small, with just a handful of potential isotopes being subject to direct labeling modalities and showing a clean palette of useful electron emissions. ^77^Br represents an attractive radionuclide for small-molecule targeted radiotherapy due to its Auger emissions depositing energy over extremely short ranges, resulting in intense and highly localized cytotoxicity. Such emissions are most effective when decay occurs in close proximity to the cellular DNA, where clustered and difficult-to-repair lesions can be generated. Additionally, ^77^Br is particularly well suited to small molecules directed against targets operating at the DNA interface or within the DNA damage response machinery. In the case of DNA-PK inhibitors, radiobromination of DNA-PK inhibitors could, in principle, combine pharmacologic inhibition of NHEJ with subcellular delivery of Auger emitters in the immediate vicinity of DNA, thereby increasing the probability of irreparable local damage while minimizing irradiation of more distant tissues.

Among radioiodine Auger emitters, both ^123^I and ^125^I are attractive for covalent incorporation into small-molecule inhibitors due to desirable emission profiles and enabling highly localized energy deposition post-decay. However, ^123^I offers several important practical and translational advantages: it is a cyclotron-produced radionuclide, has a more manageable half-life of about 13.2 h, and emits mainly one gamma photon at approximately 159 keV, which is well suited for SPECT imaging. This supports a theranostic strategy in which target engagement and biodistribution can be monitored during Auger-based radiobiologic effect [25,34]. In contrast, ^125^I (t½ ~57 d) has a splendid Auger-electron and Conversion-electron barrage, due to its electron-capture decay, its much longer half-life, which can be advantageous for in vitro preclinical work due to logistics, and prolonged experimental observation that can pose a problem in vivo when thinking about redistribution, complicating dosimetry, waste management, and prolonging whole-body activity. Additionally, it has no usable photon emissions, which means no potential live monitoring of the accumulation and biodistribution (BD).

^211^At is an especially attractive radionuclide for targeted radiotherapy when conjugated to small molecules that target DNA-associated targets, due to high linear energy transfer (LET) and short tissue range of its α-particles. Being a halogen, it also has the possibility of direct covalent incorporation into organic scaffolds through astatination chemistry. Its physical half-life of about 7.2 h is a favorable compromise, being long enough to allow the logistics chain, production, radiolabelling, quality control, and administration, yet short enough to reduce prolonged whole-body radioactive burden compared with longer-lived therapeutic radionuclides, while also being compatible with faster kinetic vectors, such as small molecules. Additionally, ^211^At is cyclotron-produced, most commonly through the ^209^Bi(α,2n)^211^1At reaction, which makes it one of the more practical α-emitters for translational radiopharmaceutical development [35,36]. Together with a very high cytotoxic effect of α-particles over a few cell diameters, ^211^At is particularly well suited for treating isolated tumor cells or micrometastases [37,38].

On the other hand, ^211^At presents important limitations. The biggest challenge is the variable in vivo stability of the C–At bond, where astatinated aryl compounds may undergo deastatination, an issue that can be more pronounced than the analogous deiodination. The astatine–carbon bond is chemically less robust and susceptible to oxidative cleavage; consequently, its successful application depends strongly on an optimized astatination chemistry, careful molecular design, and better access to optimized production infrastructure, which is constantly being enhanced [39]. Alternatively, Astatine shows metallic properties as well, which may be utilized for chelation chemistry, at the expense of a bulkier addition to the original vector, and possible extra development and optimization steps [40,41,42].

Following docking analysis, MD is often applied to obtain a more realistic evaluation of the predicted protein–ligand complex. Unlike docking, which typically provides a limited structural snapshot, MD allows the complex to be examined as a dynamic system evolving over time. This is especially important because ligand binding is influenced by continuous atomic motion and by the flexibility of both the receptor and the ligand. Recent reviews describe this transition as a movement from relatively static structure-based approaches toward dynamic simulations that better represent ligand–target recognition in biological systems [43,44].

In this context, MD is a valuable tool as it captures comprehensive biomolecular behavior with good temporal resolution, making it possible to assess the stability of a docked complex throughout the simulation [44]. Additionally, MD explicitly accounts for structural flexibility and entropic effects, which can improve the estimation of the thermodynamic and kinetic aspects of drug–target binding [45]. Previous work has also shown that MD-based approaches can support drug discovery by refining virtual screening results, identifying cryptic or allosteric binding sites, and improving the interpretation of binding energies [43,46]. Thus, MD is regarded as an essential step following docking as a validating phase for stability and biological development of the proposed ligand–receptor complex in a dynamic environment.

## 2. Materials and Methods

### 2.1. Protein Structure Prediction Using AlphaFold2

Isoform 1 of human DNA-PKcs, corresponding to NCBI RefSeq NP_008835, was selected for structural modeling due to its canonical, full-length 4128–AAs sequence that contains the complete HEAT-repeat solenoid architecture, FAT domain, kinase catalytic core, as well as the C-terminal FATC region essential for its enzymatic activity.

The first structural description of human DNA-PKcs was obtained using a full-length protein, 4128 AAs, revealing its large HEAT-repeat, helix-turns-helix motifs, and ring-like architecture that permits the protein to take a hollow ring shape [47].

The amino acid sequence of the human DNA-PKcs subunit isoform 1, encoded by PRKDC, was retrieved from the National Center for Biotechnology Information (NCBI) protein database (Reference Sequence: NP_008835), being downloaded in FASTA format and used without any truncation or manual modification for structure prediction using AlphaFold2. Due to the developers’ open-sourcing it, we were able to use AlphaFold2 through virtual access on Google Colab [48,49,50], and we would like, through this route, to acknowledge everyone who developed and made this tool available for research [51,52,53]. The tool was quite easy to use as we introduced our AA sequence, and, using Python 3 runtime type, H100 GPU, and the high-RAM setting. As in our first attempts, the need for a higher memory allocation was crucial for the program to fully be able to predict this large protein.

### 2.2. Molecular Docking

**Figure 1:**
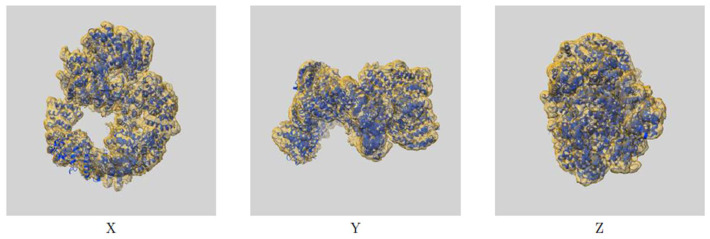
7OTW DNA-PKcs map-model overlay https://www.rcsb.org/structure/7OTW, accessed on 10 February 2026.

For the experimentally determined DNA-PK protein, we chose model 7OTW (Figure 1), obtained from RCSB PDB. The experimental method was electron microscopy (EM), and the reasons for choosing this specific item were the good resolution of 2.99 Å, the manageable number of 3D unresolved AAs gaps, 17 gaps ranging from 2 to 204 AAs (median = 8), the absence of gaps inside our critical kinase domain that we later used for the MD, as well as the presence of AZD7648 inhibitor already in the structure, highlighting the binding pocket [54]. Additionally, this model had been used before for docking studies by Liang et al. [55]

In order to be able to run the docking simulation, the protein model needs to be complete. Thus, all the gaps from EM had to be filled with the YASARA homology model. In order to do that, we loaded the protein in YASARA and identified each gap’s end AA position numbers while filling in the gap with the missing sequence. The missing sequences were obtained from UniProt.org. After filling the gaps and simulating the structure of the missing regions based on already known similar AA arrangements, we optimized each loop to make sure that the simulated structures were physically plausible. Loop optimization is important due to its capacity to improve backbone dihedrals, closure geometry, steric clashes, side-chain rotamers, local non-covalent network, and overall local energy [56,57,58,59].

Another important prerequisite for the docking analysis is the energy minimization step. Its main role is to ensure that the protein and ligand start the docking process from a physically reasonable state, with no overlaps or sensible H-bonds. That ensures that docking results are not dominated by unwanted artifacts of model building. Practically, energy minimization moves each atom’s coordinates “downhill”, from an energy point of view, so that steric clashes are removed, bonds are relaxed, and local H-bond geometry and side-chain rotamers are improved [60]. This step is quite relevant for the docking process, as once initiated, docking typically assumes that the receptor is in its most correct version, and it automatically jumps to testing ligand poses. An unminimized protein can lead to wrong scoring functions and even docking search, avoiding otherwise correct poses.

Thus, protein preparation workflows are very important, and should include steps like adding hydrogens to the protein, optimizing H-bonds, protonation fine-tuning (pH 7.4), steepest-descent minimization for removing clashes, followed by simulated-annealing minimization to reach a stable minimum before true virtual screening [61,62].

Molecular docking simulations were conducted to predict the binding affinities and molecular interactions of DNA-PK inhibitors. The cryo-EM structure of human DNA-PKcs in complex with the inhibitor AZD7648 was retrieved from the RCSB PDB database (PDB ID: 7OTW; 2.99 Å resolution). The target structure was prepared for docking using YASARA Structure [63] by adding missing hydrogen atoms at physiological pH (7.4), optimizing the hydrogen-bonding network, correcting structural inconsistencies, and performing energy minimization of the protein–ligand complex with the YASARA2 force field. All water molecules were removed prior to docking. To validate the docking protocol, the co-crystallized ligand was removed and then re-docked into the binding pocket. The predicted pose of the reference ligand was subsequently superimposed onto its original conformation, and the root-mean-square deviation (RMSD) was calculated.

Virtual ligand libraries containing DNA-PK inhibitors were prepared in DataWarrior [64] by generating 3D structures, minimizing their energy using the MMFF94s+ force field, and adjusting protonation states to physiological pH. Docking experiments were carried out in YASARA v.22.5.22 software using both AutoDock Vina v1.1.2 [65] and AutoDock4 with the Lamarckian Genetic Algorithm [66]. The search space was defined as a 25 × 25 × 25 Å box centered on the co-crystallized ligand within the active site. For each ligand, 20 docking runs were performed. The use of both algorithms allowed comparison of their suitability for predicting the binding affinity and binding pose of DNA-PK inhibitors.

To explore the structural consequences of direct radio-halogenation, a focused library of potential halogenated derivatives was generated from the selected DNA-PK inhibitor scaffolds by introducing iodine or bromine atoms at chemically feasible positions, chosen on the basis of their theoretical accessibility through established direct labeling strategies, particularly electrophilic aromatic substitution and halodestannylation. Using this rationale, approximately 67 derivatives were designed, including both iodinated, brominated, and astatinated analogs. Although astatinated derivatives were initially considered in the design phase because of their translational relevance for targeted alpha therapy, they were excluded from the docking calculations, as the YASARA platform did not support astatine-containing ligands, reducing the total number of candidates to 46. Nevertheless, the iodinated counterparts were retained as informative surrogates, given the well-recognized chemical analogy between iodine and astatine in aromatic substitution chemistry, allowing for indirect assessment of the likely impact of astatination on steric accommodation and binding behavior.

Each halogenated derivative was subjected to molecular docking using 20 docking runs per compound, and the three highest-ranked poses were retained for visual inspection. Final pose selection was not based exclusively on docking score, but also on qualitative interaction analysis within the binding pocket. More specifically, the best-ranked pose showing the most plausible binding mode and absence of unfavorable contacts or steric clashes was selected for further consideration. Compounds whose top poses displayed unfavorable interactions incompatible with stable target engagement were excluded from subsequent interpretation. This filtering step was applied to reduce the likelihood of overinterpreting formally high-scoring but structurally implausible docking solutions, yielding a number of 46 viable structures.

### 2.3. Molecular Dynamics

MD simulations were performed on the ligand–protein complexes of the ligands that were considered clinically relevant after molecular docking. For the reference system, we performed one simulation with the ligand-free (apo) structure as a negative control and one simulation with the co-crystalized ligand as a positive control.

In order to reduce computational cost, molecular dynamics simulations were performed on a truncated construct of DNA-PKcs, comprising the PI3K/PI4K catalytic domains (3722–4053 AA sequences), the G-loop (3728–3734 AA sequences), the catalytic loop (3919–3927 AA sequences), the activation loop (3939–3964 AA sequences), and the FATC domain (4096–4128 AA sequences). All of the other remaining domains were excluded. The domain boundaries were confirmed using UniProt (accession: P78527) [67]. This effectively reduced system size from 4128 AAs to 406 AAs while still preserving the full functional kinase domain. In order to neutralize artificial charges, the N- and C-terminal ends were capped with ACE and NHE groups, respectively. To further mimic the protein’s native conformation, the first and last six amino acids, including the aforementioned terminal ends, were restrained, using the “Spring to current position” tool, with a force of 35 N/m.

Simulations were conducted for a total duration of 150 ns, with snapshots being taken every 250 ps. The simulated systems were neutralized by adding Na^+^ and Cl^-^ ions to a final concentration equivalent to 0.9% NaCl. Energy minimizations were performed using the steepest descent method and simulated annealing approaches to remove potential steric clashes; the simulation volume was cubic, defined by periodic boundary conditions. The following force fields were used: AMBER14 for the protein, GAFF2 and AM1BCC for ligand atoms, and TIP3P for water molecules. Van der Waals interactions were computed with a 9 Å cutoff, while long-range electrostatic interactions were calculated using the particle mesh Ewald method without a cutoff. Simulations were maintained at 298 K and 1 atm, corresponding to the NPT ensemble. The equations of motion were integrated every 5 fps for non-bonded interactions and every 2.5 fps for bonded interactions. YASARA Structure was used to conduct the MD simulations and the binding free energy estimations [63]. The post-docking binding energy calculations were based on the MM-PBSA method, excluding the entropic contribution, using the YASARA md_analyzebindenergy.mcr macro.

## 3. Results

### 3.1. AlphaFold2 Prediction

**Figure 2:**
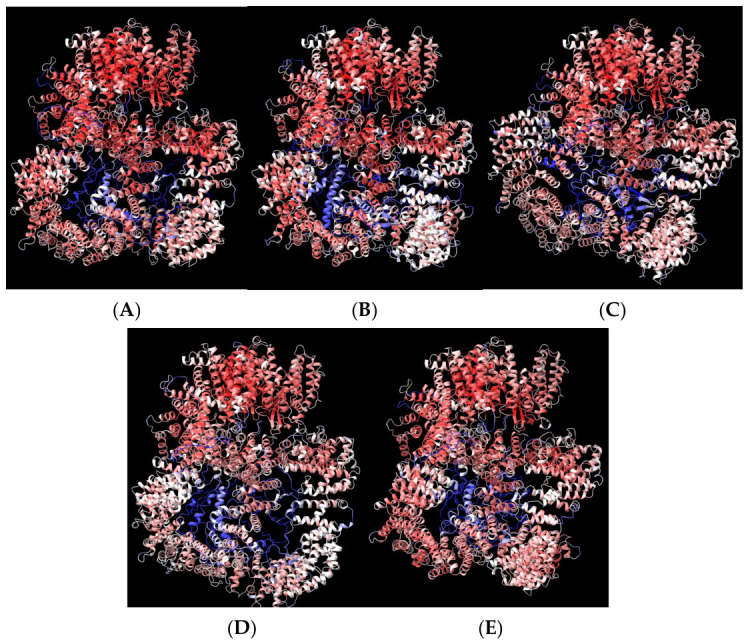
AlphaFold2 DNA-PK-predicted variants from the best to the worst confidence. (**A**) Rank 1; (**B**) Rank 2; (**C**) Rank 3; (**D**) Rank 4; (**E**) Rank 5.

**Figure 3:**
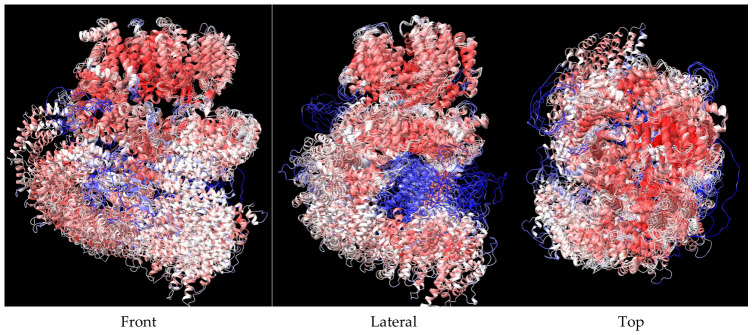
AlphaFold2 5 variants superposed. (**Front**) View from the front; (**Lateral**) View from lateral, 90° from front; (**Top**) View from the top, 90° from front.

At the end of the AlphaFold2 prediction, we obtained five renderings of the protein structure, ranked in descending order based on their confidence score (Figure 2). This score is called the Predicted Local Distance Difference Test (pLDDT) and quantifies how confident the program is about the predicted local geometry around each AA on a 0–100 scale. It is based on the local distance difference test Cα (IDDT-Cα), which is immune to superposition and analyzes the correctness of local distances [68]. The confidence levels are also color-coded with dark blue for higher confidence (90–100), light blue (70–90), yellow for low confidence (50–70), and orange–red for very low confidence (0–50). It often shows innate disordered regions, such as flexible tails, unresolved segments, or areas where the model could not choose a consistent conformation. We used the matchmaker command in the ChimeraX program to superpose and properly align the five variants; the difference, while not huge, was enough to potentially influence the end results (Figure 3). In the future, it may be interesting to use all the variants for docking studies.

When comparing two variants of one protein, even small differences in the position of atoms can influence its ligand-receptor interaction. A very easy-to-use method for quantifying the physical difference of two proteins is calculating the root-mean-square deviation (RMSD), which quantifies the average distance between corresponding atoms (often the Cα or backbone atoms), with lower values meaning closer atom positions and vice versa. For the superposed variants, the RMSD was analyzed as compared to the A variant (best-ranked), and, between all 4128 pairs, the values, in angstroms, were 12.122 for B, 16.604 for C, 3.831 for D, and 12.457 for E.

**Figure 4:**
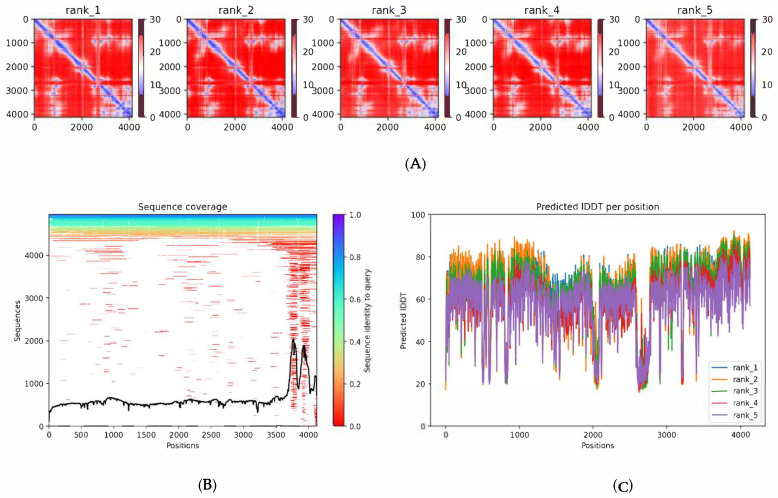
AlphaFold2 confidence metrics. (**A**) PAE matrices; (**B**) Sequence coverage; (**C**) pLDDT.

The other metrics that we got from the prediction (Figure 4) are the sequence coverage, predicted IDDT per position, and the predicted aligned error (PAE) matrices, which can help identify which areas of the predicted structure are reliable and which still have uncertainty.

On the predicted pLDDT plot, we can see a pretty consistent and reasonable prediction, around 60–80% confidence, in the N-terminal segment (approx. residues 1–600) and mid-region (≈800–2500). In contrast, residues ~601–800 and ~2401–4200 dip into the orange/red (pLDDT < 50–60) region. Such low scores often arise for intrinsically flexible or disordered loops, or regions with poor MSA coverage. Regions with lower pLDDT than 50 should be considered unreliable for detailed modeling.

The PAE matrices indicate the expected alignment errors between residue pairs, calculated as position error in angstroms from lower (blue) to higher (red). Blue areas along the diagonal indicate low inter-residue error.

**Figure 5:**
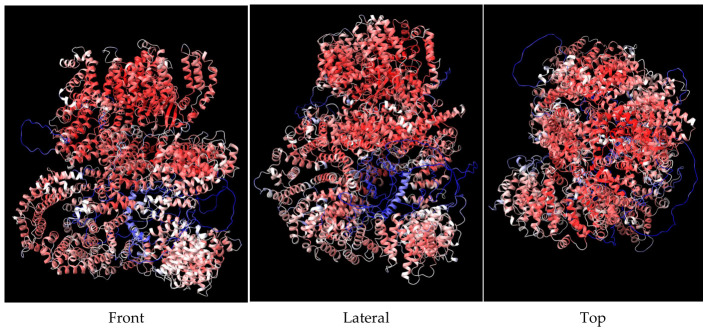
Best ranking DNA-PK variant based on the predictor algorithm. (**Front**) View from the front; (**Lateral**) View from lateral, 90° from front; (**Top**) View from the top, 90° from front.

The pLDDT score varies significantly along a protein prediction, which can be an indication of what regions the algorithm is most confident in, meaning better reliability of that zone. Thus, it is important to understand that a low pLDDT does not always indicate wrong prediction, as it might indicate a flexible segment, which can be biologically accurate. Nevertheless, we chose the protein labeled rank 1 to go forward with the docking (Figure 5), since we want to assess the reliability and robustness of the simulation program; we feel that the final decision of the program is also to be assessed.

### 3.2. Molecular Docking

**Figure 6:**
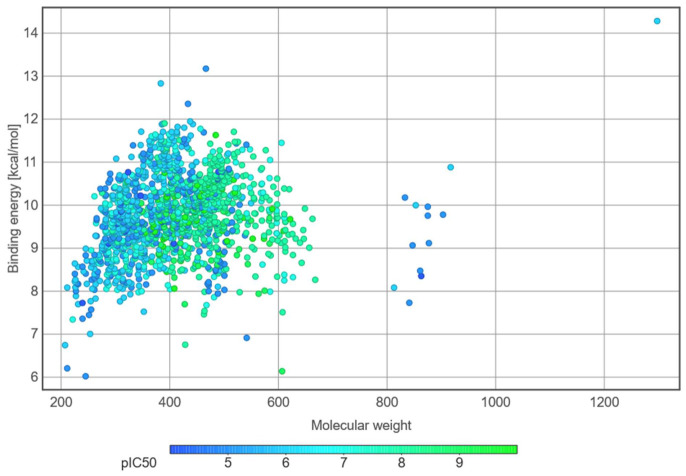
Distribution of predicted docking binding energies (absolute values in Kcal/mol) for the 1369 screened compounds, in accordance with molecular weight and pIC50 calculated from ChEMBL data.

The screening program for the 1369 compounds extracted from ChEMBL activity assays on human DNA-PK target, after filtering for compounds with only IC50 exact nanomolar concentrations, resulted in binding energies ranging from −6.022 Kcal/mol to −14.282 Kcal/mol (Figure 6). Most screened ligands represented a relatively compact molecular weight interval, predominantly between 250 and 600 Da, while their docking scores were concentrated mainly between approximately −8 and −11 kcal/mol. This indicates that the screened chemical space was centered on medium-sized, drug-like compounds, with only a limited number of higher-molecular-weight outliers.

A modest tendency toward more favorable predicted binding energies was observed with increasing molecular weight within the main cluster, although this trend was not uniform, and several compounds of comparable size still showed substantial energy variability. This can be explained through the number of interactions between larger compounds and the binding pockets, which likely contributed more to the predicted binding behavior. Overall, the distribution depicts a chemically heterogeneous inhibitor set, in which most compounds fall within a similar size range but retain a broad spectrum of predicted binding energies.

**Figure 7:**
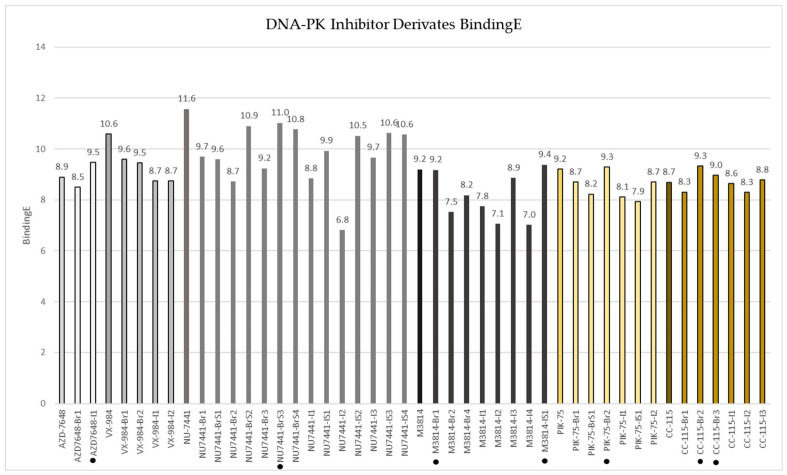
DNA-PK inhibitor radioderivates binding energies. Potential improved derivatives are marked with a dot.

**Figure 8:**
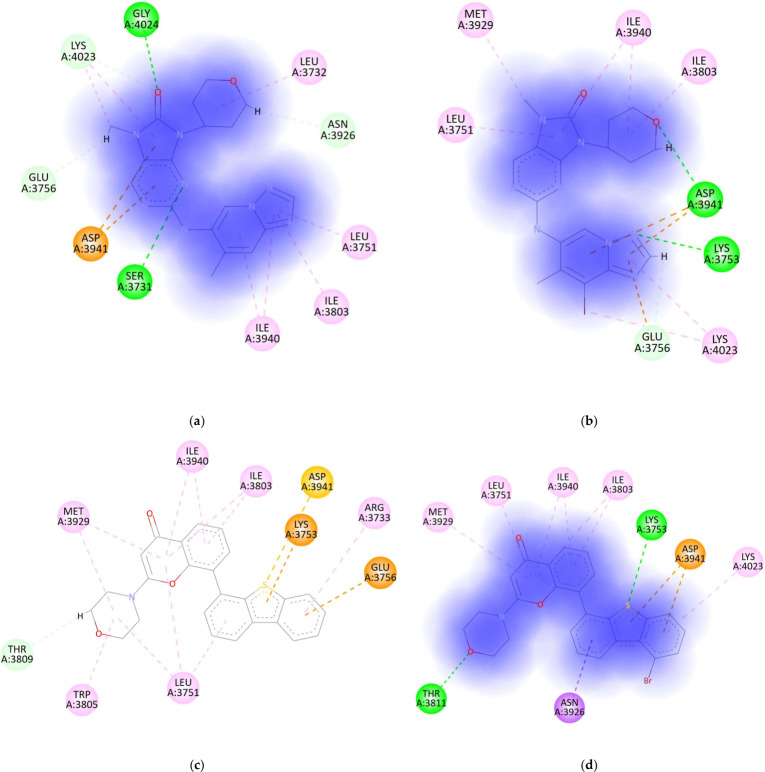

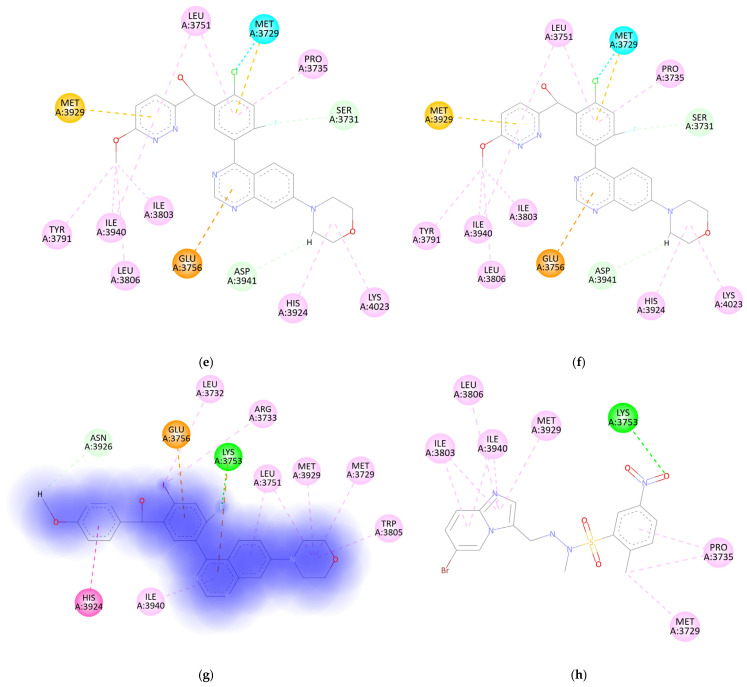

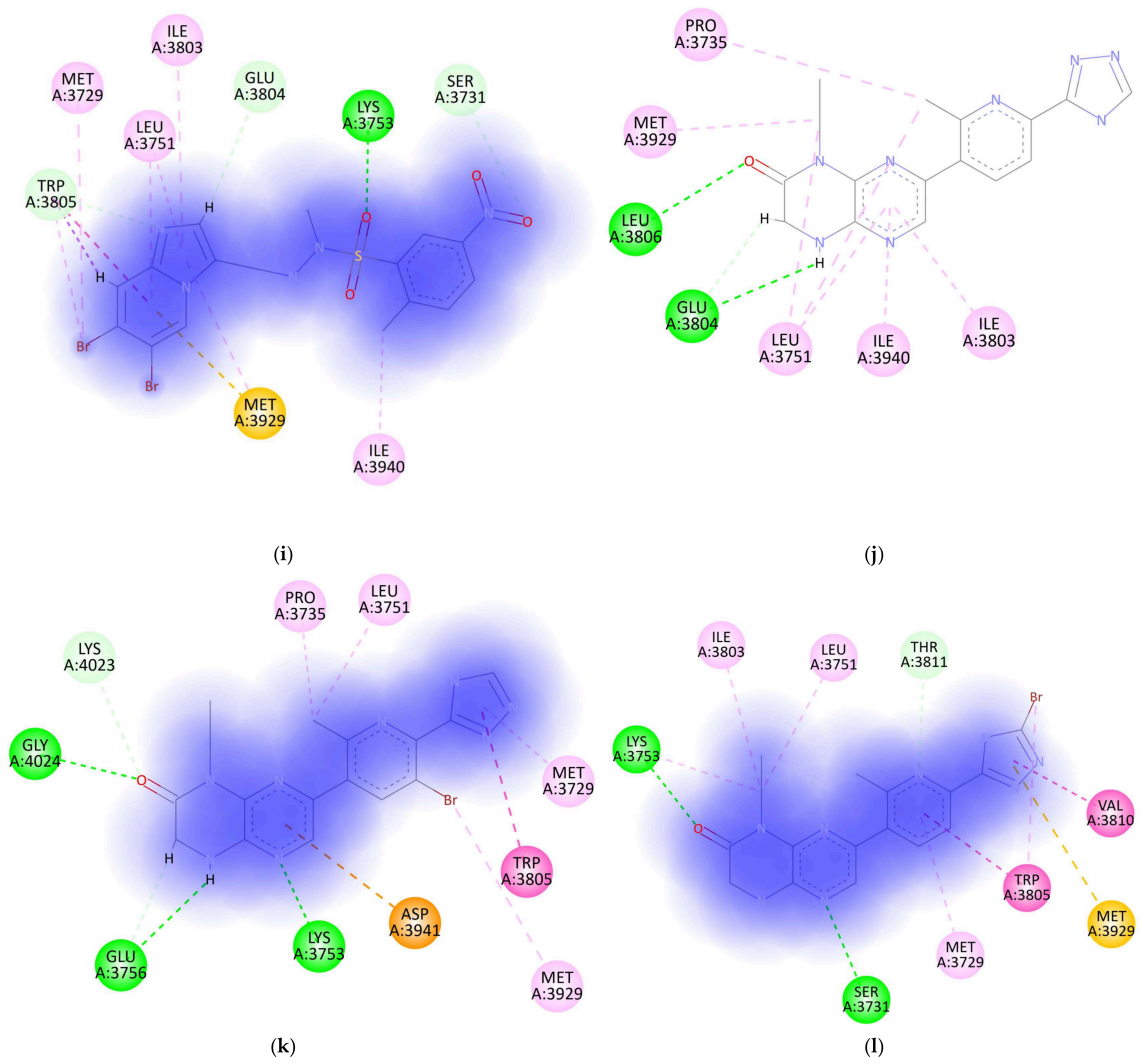
DNA-PK inhibitors molecular interactions: (**a**) AZD-7648, (**b**) AZD7648-I1, (**c**) NU-7441, (**d**) NU7441-BrS3, (**e**) M3814, (**f**) M3814-IS1, (**g**) M3814-Br1, (**h**) PIK-75, (**i**) PIK-75-Br2, (**j**) CC-115, (**k**) CC-115-Br2, and (**l**) CC-115-Br3.

Across the 46 halogenated derivatives that passed pose-quality filtering (Figure 7), the docking readout—binding energy (BE)—showed a clear and encouraging trend: most Br/I substitutions were broadly “binding-neutral”, with predicted energies remaining close to the corresponding parent scaffold, rather than collapsing because of steric incompatibility. Introducing a single halogen entity at chemically plausible positions on these DNA-PK inhibitor chemotypes seems to generally preserve the core binding mode. Within each parent series, there were also a few derivatives with clearly higher BE values, meaning more favorable due to YASARA docking protocol showing binding energies as an absolute value, suggesting that in selected positions the halogen can be accommodated in a way that strengthens shape complementarity or local noncovalent contacts without disrupting the pose (AZD7648-I1, NU7441-BrS3, M3814-Br1, M3814-IS1, PIK-75-Br2, CC-115-Br2, CC-115-Br3) (Figure 8). However, within each series, the majority of compounds showed comparable or worse binding energies.

Importantly, we did not rely on the docking score alone. From the top-ranked poses (after repeated runs per ligand), the final set reflects visual inspection to remove poses dominated by unfavorable interactions (e.g., steric clashes, implausible burial, or strained orientations), which is why some designed variants were excluded despite being formally docked. The dotted bars highlight the most compelling candidates for follow-up because they combine favorable BindingE with structurally plausible binding geometry. Thus, the dotted compounds together represent the clearest starting points for deeper refinement (e.g., MD-based stability checks, rescoring, and development of synthesis and radiochemistry optimization).

Compared with compound a, compound b appears to establish a more extended interaction network within the DNA-PK binding pocket, including an additional polar contact in the Glu3756 region and favorable contacts toward Asp3941/Lys3753, while maintaining hydrophobic packing with surrounding residues. These features may explain the improved predicted binding energy of b.

Compared with compound e, compound g may exhibit improved binding due to the replacement of chlorine with iodine, which increases steric bulk and polarizability and likely enhances hydrophobic/dispersion interactions within the lipophilic region of the DNA-PK binding pocket. The iodine substituent may also contribute to improved local complementarity while preserving key anchoring interactions, resulting in a more favorable predicted binding energy.

Compared with compound k, both l and m appear to adopt a more favorable interaction pattern within the DNA-PK binding pocket, which may explain their improved predicted binding energies. Compound k seems to rely mainly on the classical anchoring interactions around Leu3806 and Glu3804, with a comparatively less extensive engagement of the distal region of the pocket. In contrast, compound l establishes a broader interaction network, including contacts with Gly4024, Glu3756, and Lys3753, together with additional contacts toward Asp3941 and the Trp3805 region, suggesting a more strongly stabilized binding mode. Compound m also appears to bind more favorably than k, but its improvement seems to arise predominantly from better hydrophobic occupation of the distal pocket, involving residues such as Trp3805, Met3929, Val3810, Leu3751, and Ile3803, while retaining key anchoring interactions. Overall, compound l seems to display the strongest binding of the three because it combines extended polar interactions with good pocket occupancy, whereas m, although still superior to k, appears to rely more on lipophilic stabilization and therefore may be slightly less strongly bound than l.

### 3.3. Molecular Dynamics

The solute RMSD from the starting structure was monitored throughout each simulation to assess the global conformational stability of the DNA-PKcs kinase domain construct (residues 3722–4128) in complex with each of the six candidate compounds (Table 2). All systems reached conformational equilibrium within the first 30–40 ns of the simulation. The positive-control simulation of the co-crystallized inhibitor showed stable Cα RMSD values oscillating between 3.0 and 4.5 Å, with all-atom RMSD plateauing at approximately 4.7–5.0 Å.

The CC-115 and PIK-75 complexes displayed Cα RMSD values stabilizing at 4.5–5.0 Å, with all-atom RMSD converging to 5.0–5.5 Å, closely consistent with the positive control. M3814 reached a Cα RMSD plateau of 3.5–4.5 Å during the first half of the trajectory, followed by a gradual upward trend in all-atom RMSD in the final 50 ns. AZD-7648 and VX-984 exhibited non-convergent behavior, with all-atom RMSD values rising continuously to 6.5–8.5 Å over the course of the simulation. NU7441 exhibited similar behavior, reaching a Cα RMSD plateau of 3.5–4 Å, and remaining relatively stable within that range.

**Figure 9:**
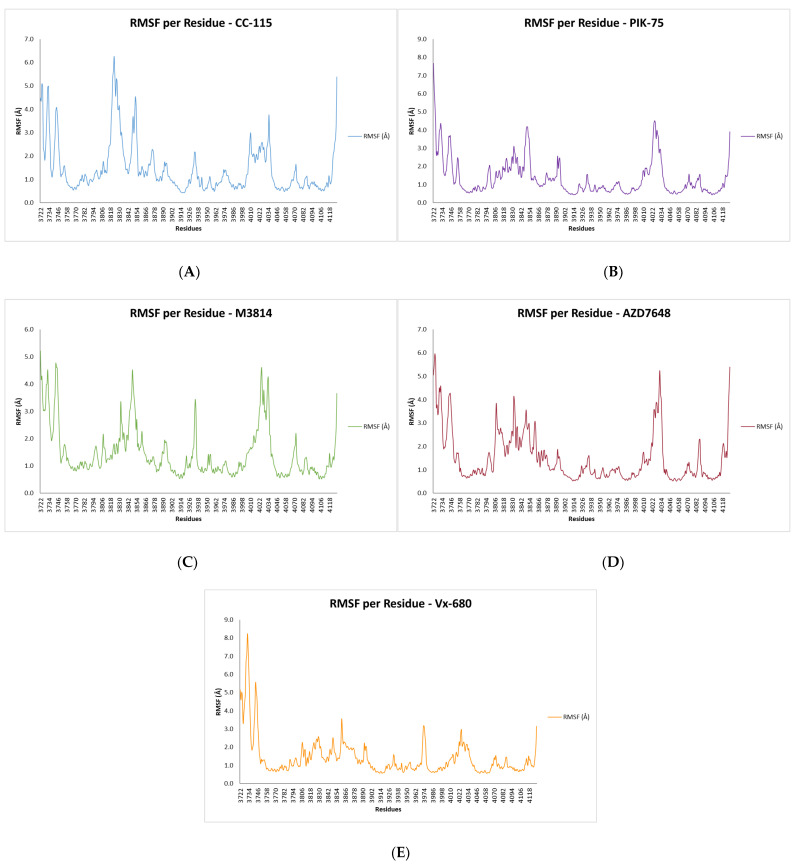
RMSF of docked ligands during the MD run, (**A**) CC-115, (**B**) PIK-75, (**C**) M3814, (**D**) AZD7648 and (**E**) VX-680.

The ligand movement RMSD, computed after superposition of each trajectory frame on the protein, was used to assess the translational stability of each compound within the binding site (Table 3). CC-115 displayed relatively stable binding throughout the 178 ns simulation, with ligand movement RMSD settling at 3.5–5.5 Å following initial equilibration and remaining within this range for the duration of the trajectory (Figure 9A). The internal conformation RMSD of CC-115 ranged from 1.1 to 2.3 Å, and the mean ligand RMSF was 1.71 Å.

PIK-75 similarly maintained relatively stable binding over the 178 ns simulation (Figure 9B). After a transient displacement to approximately 6.4 Å during the first 10 ns, ligand movement RMSD converged to 3.5–4.5 Å and remained stable for the remainder of the trajectory. Internal conformation RMSD was consistently low (0.4–2.0 Å), with one transient deviation near 80 ns. Mean ligand RMSF was simulated as 1.89 Å.

NU7441 suffered displacement in the first 20 ns and then remained at a stable RMSD of 9 Å for the rest of the simulation. Internal conformation RMSD remained at under 1 Å after an initial spike to 1.2 Å.

M3814 remained associated with the binding site throughout the simulation, with ligand movement RMSD of 2.0–3.5 Å during the first 120 ns, followed by a progressive increase to 6.0–9.0 Å in the final 50 ns of the simulated trajectory (Figure 9C). Internal conformation RMSD ranged from 1.0 to 2.4 Å, and the mean ligand RMSF was 2.75 Å.

AZD-7648 maintained partial binding-site association during the first 20 ns of the simulation, with ligand movement RMSD of ~3 Å, before undergoing an abrupt transition to values of 50–60 Å, indicating complete dissociation from the binding site (Figure 9D). Internal conformation RMSD remained low throughout (0.3–1.3 Å), and mean ligand RMSF was 19.56 Å. VX-984 dissociated rapidly, with ligand movement RMSD rising to approximately 35 Å by 55 ns and reaching 40–68 Å for the remainder of the simulated trajectory (Figure 9E). Mean ligand RMSF was 28.39 Å. Internal conformation RMSD prior to dissociation ranged from 0.5 to 3.7 Å.

**Figure 10:**
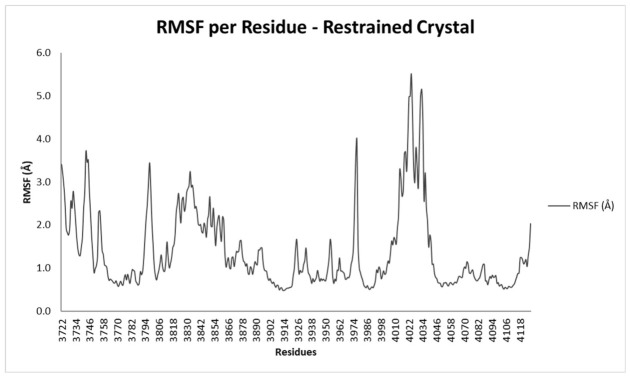
RMSF of the Restrained Crystal during the MD run.

Per-residue Cα RMSF was calculated to identify regions of differential flexibility across all simulated systems (Figure 10). The glycine-rich loop (G-loop, residues 3728–3734) was the most flexible region in all simulations. In the positive-control simulation, the mean G-loop RMSF was 2.29 Å. Among the test compounds, mean G-loop RMSF values were 3.49 Å for CC-115, 3.55 Å for PIK-75, 3.64 Å for M3814, 3.88 Å for AZD-7648, 6.37 Å for VX-984, and 2.91 Å for NU7441 (Table 3).

The catalytic loop (residues 3919–3927) and activation loop (residues 3939–3964) showed uniformly low RMSF values across all systems (0.6–1.5 Å). FATC domain residues (4096–4128) presented mean RMSF values of 0.85–1.35 Å. Elevated RMSF at the N- and C-terminal boundaries of the construct (residues 3722–3727 and 4122–4128) was confined to the terminal six residues at each end, consistent with the artificial capping groups of the truncated construct.

## 4. Discussion

When analyzing the pLDDT as an indicator of AlphaFold2’s reliability of prediction beyond search algorithms, the accuracy of docking depends on how accurately the binding energy is predicted, including intermolecular interactions, solvation, and conformational changes. Because exact free-energy calculation would be too expensive for routine docking studies, scoring functions use simplified mathematical models that can be classified into four types: force-field/physics-based, empirical, knowledge-based, and machine-learning-based scoring. Physics-based functions sum bonded and non-bonded terms such as Van der Waals, electrostatics, hydrogen bonding, and solvation; empirical functions fit weighted interaction terms to experimental binding data; knowledge-based functions derive statistical potentials from known protein–ligand structures, often using the inverse Boltzmann relation, where frequently observed atom-pair contacts get marked as favorable; machine-learning models move beyond fixed linear formulas and learn non-linear relationships from large datasets [4,69,70].

A key limitation of standard docking studies is that real biomolecules are not static. Ligands adopt multiple conformations, and proteins usually change their shape upon binding. This can be described in terms of induced fit and conformational selection. Induced fit means the protein adjusts after ligand approach, while conformational selection means the protein already exists as an ensemble of states and the ligand binds to one of them, shifting the population. This is key because the rigid-docking-obtained information shows a rather rough approximation. To handle flexibility, several strategies, such as soft docking, side-chain flexibility, molecular relaxation, and ensemble docking, were developed. Ensemble docking is especially important due to its replacing the single-structure approach of the receptor with a population-based one, which is closer to modern structural biology understanding [3,4].

Another core limitation is how the presence of water, entropy, and dynamics is being integrated. Docking often performs reasonably well for pose generation but still needs improvement for precise binding-affinity prediction, mainly due to the solvent being treated too crudely, hydrogen-bond networks being oversimplified, and the real dynamics of the system needing more complexity. Most docking algorithms neglect explicit water or include it only implicitly, yet water molecules can mediate key contacts and reshape the whole interaction network. Entropy is also difficult to accurately represent due to the binding changing conformational freedom in both ligand and protein. This is why docking scores should not be confounded with exact physical binding free energies, but are rather useful approximations, utilized for ranking and hypothesis generation, but not sound thermodynamic descriptions [3,4,71].

The RMSD of the truncated domain averaged 4 Å in all MD simulations, which suggested kinase domain structural instability, even with 30 N/m restraints. These results suggest that the FAT domain might have a fundamental role in maintaining the stability of the binding pocket. Although performing an MD simulation on the full protein seems unfeasible on most systems, further studies should also include the FAT domain, acting as a structural scaffold to the kinase catalytic domains.

Our PDB included AZD7684 as the co-crystalized ligand, which we used as a positive control. The positive control ligand RMSD after superposing on the receptor suggested the ligand underwent transient departures from the native binding model before a final transition to a more energetically stable conformation.

The redocked AZD7684 and the docked VX984 experienced spontaneous unbinding due to unfavorable binding free energy values, which were later confirmed by the MM-PBSA calculations. This spontaneous unbinding may have been caused by the overall instability of the truncated domain and unfavorable computed intermolecular interactions. PIK75 exhibited a conformational profile similar to the native control, yet demonstrated a less stable conformation, and migrated from the binding pocket at the 150 ns simulation time. M3814 remained stable at first, but gradually migrated away from the binding pocket, reaching a constant RMSD of >5 after 100 ns. Accordingly, these simulations do not support accurate affinity ranking or quantitative thermodynamic conclusions.

All MM-PBSA calculations averaged positive binding free energies, indicating that, although some ligands remained structurally stable relative to the highly flexible system, all systems were thermodynamically unstable, failing to recreate physiological binding conditions.

Simulations that included 30 N/m restraints on all protein atoms showed nearly identical results, further showing that restraints alone were unable to compensate for the missing structural domains.

To conclude, given that the ligand superposition RMSD values were combined with the unstable MM-PBSA binding free energy values, this truncated system proved to be thermodynamically unstable and unreliable for simulating accurate ligand binding free energy and binding domain conformation. Further research should include other domains of the DNA PKcs enzyme for a more thermodynamically stable system and a physically stable binding pocket.

Overall, the present study supports the value that a tiered computational workflow—which includes AI-assisted receptor modeling, large-scale docking, and focused derivative screening—can have in prioritizing radiopharmaceutical design hypotheses before experimental synthesis. Thus, future docking studies remain highly relevant for tracer design, not as stand-alone proof of efficacy, but as a rational filter for identifying modification patterns that preserve target engagement while accommodating desired alterations. This is especially important for DNA-PK inhibitors, due to DNA-PKcs being a central component of the NHEJ machine and being recruited to DNA double-strand break sites, making it an attractive pharmacologic partner for targeted radiotherapy (TRT) and a plausible vector class for targeted radionuclide therapy. Recent reviews have explicitly highlighted DNA repair inhibitors, including DNA-PK inhibitors, as promising targets or partners for TRT, while experimental studies continue to show that DNA-PK inhibition can strongly enhance radiation response [17,72,73].

At the same time, the translational value of these compounds depends on substantially deeper experimental characterization. Docking-compatible derivatives must still be validated with respect to synthesis feasibility, radiolabeling chemistry, target affinity, intracellular trafficking, nuclear localization, DNA-proximal residence, metabolic stability, and therapeutic index. The same caution applies to the receptor models used for prediction: AlphaFold2 has transformed access to structural hypotheses, but its performance remains limited in dynamic systems, ligand-induced conformational states, flexible regions, and contexts where binding-site geometry is sensitive to cofactors or alternate protein states. Recent work has shown that AlphaFold-based models can support virtual screening, underscoring the need for better receptor preparation and more realistic simulation pipelines. Thus, the next phase of this field will likely rely not only on better ligands, but also on better prediction tools—including refined AlphaFold-derived structures, improved treatment of receptor flexibility, and more predictive molecular simulation frameworks that can bridge the gap from informative binding to true radiopharmaceutical behavior in biologic systems [74,75,76].

## Figures and Tables

**Table 1:** List of several studied DNA-PK inhibitors relevant to our study.

Molecule	Name	IUPAC Name
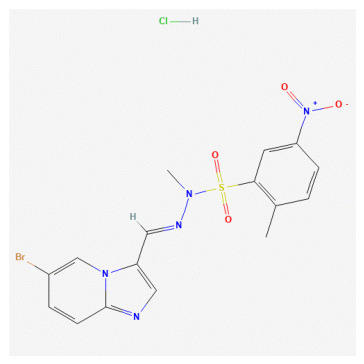	PIK-75 HCl	*(N-[(E)-(6-bromoimidazo[1,2-a]pyridin-3-yl)methylideneamino]-N,2-dimethyl-5-nitrobenzenesulfonamide;hydrochloride)*
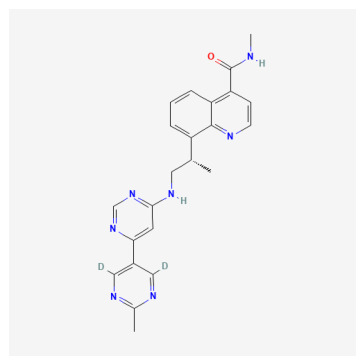	VX-984	*(8-[(2S)-1-[[6-(4,6-dideuterio-2-methylpyrimidin-5-yl)pyrimidin-4-yl]amino]propan-2-yl]-N-methylquinoline-4-carboxamide)*
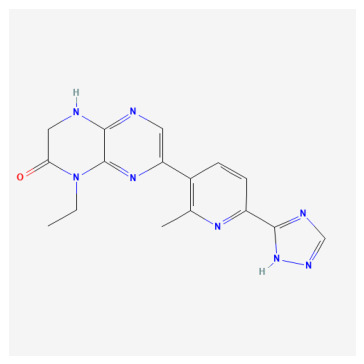	CC-115	*(5-ethyl-3-[2-methyl-6-(1H-1,2,4-triazol-5-yl)-3-pyridinyl]-7,8-dihydropyrazino[2,3-b]pyrazin-6-one)*
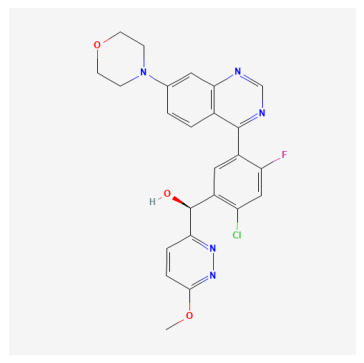	M3814 (Peposertib)	*((S)-[2-chloro-4-fluoro-5-(7-morpholin-4-ylquinazolin-4-yl)phenyl]-(6-methoxypyridazin-3-yl)methanol)*
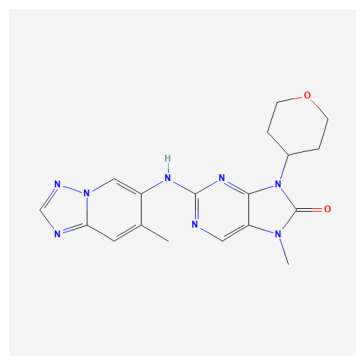	AZD-7648	*(7-methyl-2-[(7-methyl-1,2,4]triazolo[1,5-a]pyridin-6-yl)amino]-9-(oxan-4-yl)purin-8-one)*
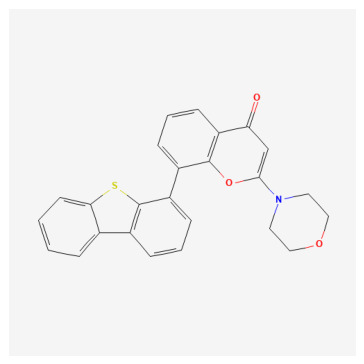	NU-7441	*(8-dibenzothiophen-4-yl-2-morpholin-4-ylchromen-4-one)*

**Table 2:** Summary of MD simulation stability metrics for the five candidate DNA-PKcs radiosensitizers and the positive-control simulation.

Compound	Duration (ns)	Cα RMSD Plateau (Å)	Ligand Movement RMSD (Å)	Ligand Conf. RMSD (Å)	Ligand RMSF Mean (Å)	Binding Outcome Relative to Control
Positive control	170	3.0–4.5	2.0–4.5	0.4–1.9	—	Stable (reference)
CC115	178	4.5–5.0	3.5–5.5	1.1–2.3	1.71	Stable
PIK75	178	3.5–5.0	3.5–4.5	0.4–2.0	1.89	Stable
M3814	170	3.5–4.5	2.0–9.0	1.0–2.4	2.75	Partial drift
AZD7648	165	4.5–5.0 (rising)	~10–18 → ~60 (post ~110 ns)	0.3–1.3	19.56	Dissociated
VX984	150	3.5–5.0 (rising)	~40–68 (post ~55 ns)	0.5–3.7	28.39	Dissociated
NU7441	150	3.0–4.0	7.5–9	0.2–1	3.18	Dissociated
Negative control (APO)	150	2.5–3.5	-	-	-	Stable (reference)

**Table 3:** Mean Cα RMSF (Å) for key functional regions of the DNA-PKcs kinase domain across all simulated systems.

System	G-Loop (3728–3734)	Catalytic Loop (3919–3927)	Activation Loop (3939–3964)	FATC Domain (4096–4128)
Positive control	2.29	0.98	0.90	0.85
CC115	3.49	0.69	0.72	1.17
PIK75	3.55	0.80	0.74	0.96
M3814	3.64	0.98	0.93	1.03
AZD7648	3.88	0.81	0.79	1.35
VX984	6.37	0.86	0.86	1.05
NU7441	2.91	0.97	1.08	1.26
Negative control (APO)	2.91	1.42	1.40	1.54

## Data Availability

The original contributions presented in the study are included in the article, further inquiries can be directed to the corresponding author.

## Abbreviations

The following abbreviations are used in this manuscript:

DNA-PK	DNA-dependent protein kinase
DNA-PKcs	DNA-PK catalytic subunit
NHEJ	Non-homologous end joining
DSB	Double-strand breaks
HR	Homologous recombination
MD	Molecular dynamics
AA	Amino acid
pLDDT	Predicted Local Distance Difference Test
IDDT-Cα	Local distance difference test Cα
RMSD	Root-mean-square deviation
PAE	Predicted aligned error
EM	Electron microscopy
TRT	Targeted radiotherapy
